# Enrichment of sulphate-reducers and depletion of butyrate-producers may be hyperglycaemia signatures in the diabetic oral microbiome

**DOI:** 10.1080/20002297.2022.2082727

**Published:** 2022-06-03

**Authors:** Camilla Pedrosa Vieira Lima, Daniela Corrêa Grisi, Maria Do Carmo Machado Guimarães, Loise Pedrosa Salles, Paula de Castro Kruly, Thuy Do, Luiz Gustavo Dos Anjos Borges, Naile Dame-Teixeira

**Affiliations:** a School of Health Sciences, University of Brasilia, Brasilia, Brazil; b School of Dentistry, University of Leeds, Leeds, UK; c Microbial Interactions and Processes Research Group, Helmholtz Centre for Infection Research, Braunschweig, Germany

**Keywords:** Oral microbiome, hyperglycaemia, blood glucose, salivary glucose, salivary pH, diabetes mellitus, periodontitis, 16S rRNA sequencing

## Abstract

**Objectives:**

This study aimed to investigate oral microbial signatures associated with hyperglycaemia, by correlating the oral microbiome with three glycaemic markers. Potential association between clinical parameters and oral bacterial taxa that could be modulating the hyperglycaemic microbiome was also explored.

**Methods:**

Twenty-three individuals diagnosed with type 2 Diabetes Mellitus (T2D) and presenting periodontitis were included, as well as 25 systemically and periodontally healthy ones. Fasting blood glucose, glycated haemoglobin, salivary glucose, periodontitis classification, caries experience and activity and salivary pH were evaluated. The V4 region of the *16S rRNA* gene was amplified from total salivary DNA, and amplicons were sequenced (Illumina MiSeq).

**Results:**

Hyperglycaemia was correlated with proportions of *Treponema, Desulfobulbus, Phocaiecola* and *Saccharimonadaceae. Desulfobulbus* was ubiquitous and the most enriched organism in T2D individuals (log2FC = 4). The *Firmicutes/Bacteroidetes* ratio was higher at alkali salivary pH than acidic pH. In the network analysis, *Desulfobulbus* was clustered in a negative association with caries-associated and butyrate-producing bacteria.

**Conclusion:**

The salivary microbiome is shaped by systemic hyperglycaemia, as well as changes in the salivary pH, which may be linked to local hyperglycaemia. The enrichment of predictive biomarkers of gut dysbiosis in the salivary microbiome can reflect its capacity for impairment of hyperglycaemia.

## Introduction

Several oral manifestations of Diabetes Mellitus (DM) can be explained by the hyperglycaemia state that directly favours the enrichment of microbial pathogens, promoting damage of cellular function, and consequently, local inflammatory responses. This occurs due to interactions between the increased concentration of advanced glycation end-products and the increased proinflammatory cytokine levels. A well-established oral manifestation of DM is periodontitis, which may also impair the systemic glycaemia control [1,2]. A reduced salivary flow is also commonly observed in individuals suffering from this metabolic disorder [3,4]. The oral health is deprecated, essentially when the glycaemic levels are uncontrolled [5,6]. The poor glycaemic control can make adults with type 2 DM (T2D) more prone to dental caries, although the reasons behind this association are not yet explained [7]. A potential hypothesis is that the hyperglycaemia may increase the glucose levels in the saliva of patients, changing the oral microbial environment and promoting salivary acidification [8].

A growing body of literature recognizes the importance of salivary glucose as a biomarker of blood glucose levels [9–12]. Salivary glucose may be accountable for reducing the pH of the oral cavity, since oral bacteria can use glucose as a substrate in fermentative pathways, releasing acids as final metabolites. If these changes in the availability of metabolic substrates linger, the so-called ‘dynamic stability stage’ of the oral microbiome can be lost [13]. The acidification would facilitate acidogenic bacterial growth, shifting the ecological balance of the microbiota [8,14]. Furthermore, individuals with uncontrolled DM frequently present ketoacidosis increasing the ketone bodies (acetone, acetoacetic acid, and β-hydroxybutyric acid) in blood and urine [15], and probably in saliva. The potentially altered pH [4] can represent a selective pressure over the diabetic oral microbiome. If the pH-balance of the microbial community is disrupted by severe environmental pressures, the microbiome may collapse into an ‘acidogenic stage’ (increase in the acidogenic microorganisms) that initiates dental caries or into an ‘’inflammatory stage” (increase of inflammophilic anaerobic microorganisms) leading to periodontitis [13].

The impact of DM on the salivary microbial biodiversity has been investigated [14,16–19]. *Bacteroidota* and *Proteobacteria* are enriched in the salivary microbiome of DM patients, somehow reflecting the pattern seen in the gut microbiome [20], and suggesting a potential correlation of gut and oral microbiomes in diabetic subjects. Indeed, the imbalance observed in the gut microbiota might be a main contributor of local and systemic diseases [18,21]. Microbial communities along mucosal surfaces throughout the digestive tract are hypothesized as risk factors for impaired glucose regulation. Since some gastric bacteria are introduced through the oral cavity, it is possible that a decreased salivary pH due to hyperglycaemia may act as a filter to inhibit replenishment of gastric *Bacteroidota*, while more easily transmitting gastric *Firmicutes* [8]. This can also be explained by the communication through secondary metabolites of different microbiota of the human body [22]. Nevertheless, it is not yet clear if dysbiosis in the oral microbiome is a typical feature of hyperglycaemia and a potential contributor to progression of hyperglycaemia itself. Microbial metabolites from the oral cavity could serve as crosstalk mediators between host and microbiome, impacting glucose metabolism. So far, the oral microbial signatures associated with hyperglycaemia, as well as its oral manifestations, are still undetermined.

The oral dysbiosis–diabetes relationship is to be elucidated. A fundamental need is understanding how the oral microbiome shifts from homeostatic to dysbiotic condition, altering the oral health status of DM patients. Understanding this process would allow more efficient treatments for oral manifestations of DM. This study aimed to investigate oral microbial signatures associated with systemic and local hyperglycaemia, by correlating the oral microbiome with three glycaemic biomarkers (glycate haemoglobin (HbA1c), fasting blood glucose (FBG) and salivary glucose). We also aimed to explore a potential association between biological markers and oral bacterial taxa that could be modulating the hyperglycaemic microbiome, such as the salivary pH, T2D and periodontitis diagnosis, and levels of dental caries.

## Subjects and methods

### Ethics

The study was approved by the Research Ethics Committee of the School of Health Sciences of the University of Brasilia (process number 87962818.4.0000.0030) in accordance with the declaration of Helsinki. All patients signed a formal consent form and received basic dental treatment. Healthy participants received oral hygiene instruction and professional prophylaxis.

### Study design, setting and participants

This study was nested in a cross-sectional study [23], and it was performed and reported following the STROBE checklist [24]. Eligible individuals (>18 years old) were enrolled in the Diabetes Dental Clinic at the University Hospital of Brasilia (Federal District, Brazil). Patients were recruited from June 2018 to March 2020.

Individuals with and without a diagnosis of T2D were included in order to ensure a broad range of glycaemic levels. Cases of T2D were defined through a previous medical diagnosis. All patients in this group were using hypoglycaemic medication, either insulin or another hypoglycaemic drug. Only individuals diagnosed with any level of periodontitis were included in this group to assure its homogeneity (interdental clinical attachment loss ≥3 mm detectable at ≥2 non‐adjacent teeth) [25]. Another group of systemically and periodontally healthy individuals was included (named as no-T2D), which were selected among family members and other individuals under treatment at the university clinics. All individuals, either T2D or no-T2D, went through blood and saliva glucose levels measurements (as described below). Individuals with type 1 DM were excluded, as well as those with severe systemic comorbidities, pregnant or puerperia, transplanted patients, individuals with a history of epilepsy, or with systemic conditions that may influence the physiology of the salivary gland, such as hypothyroidism, radiotherapy or chemotherapy treatment that preceded 3 months.

Based on a pilot study [26], a minimum number of 14 samples is required to detect a correlation of 0.7 with power of 80% in an alpha of 5% (Fisher’s Z test) between bacteria taxa and clinical parameters. For a mean difference of 2 (standard deviation of 1.5 and 3.1) in the *Firmicutes/Bacteriodota* ratio between T2D and no-T2D microbiomes, a minimum of 48 samples is required, to which was added a loss rate of 10%, resulting in a sample size of 52 individuals.

### Salivary sampling

Stimulated and passive salivary flow samplings were performed in the morning (8–10 am) to minimize the effect of circadian rhythms. Individuals were asked to refrain from drinking, eating, and performing physical activities at least 2 h before salivary collection. The salivary collection was carried out for 5 min of passive drooling. Upon collection, 500 μL of the saliva samples were aliquoted into microtubes and pellets stored at −20°C until further DNA extraction and sequencing. Stimulated saliva was also collected for 5 min, while participants chewed a rubber device (1 x 1 cm, free of flavor). They were tied to a piece of dental floss so that there was no danger of swallowing by the patient during chewing.

### Glycaemic markers

Fasting blood glucose (FBG) (hexokinase method; mg/dL) and glycated haemoglobin (HbA1c) (turbidimetric inhibition immunoassay; %) tests were carried out at the university’s partner-certified centre of diagnosis (Sabin labs, Brasilia – Distrito Federal, Brazil). Salivary glucose was measured from the stimulated saliva using the Labtest Glucose liquiform® kit (Labtest Diagnóstica S.A – Minas Gerais, Brazil), according to the manufacturer’s instructions with an adaptation for the saliva volumes, as follows: after centrifugation, 150 µL of the supernatant was added to 500 µL of the kit reagent 1 (phosphate buffer 30 mmol/L, pH 7.5; phenol 1 mmol/L; glucose oxidase 12,500 U/L; peroxidase 800 U/L; 4-aminoantipyrine 290 mol/L; azide sodium 7.5 mmol/L; and surfactant). A glucose standard was added to the experiment. After homogenization and incubation at 37°C for 10 min, 250 µL of the reaction was transferred to the 96-wells plate, in duplicates, and read at 505 nm. Blood and glucose levels were analysed as continuous variables, and the salivary glucose was also categorised as high (≥0.35 mg/dL) and low (<0.35 mg/dL), according to the data distribution.

### Sucrose frequency intake

A 24-h diet recall was performed to determine the frequency of sucrose intake.

### Periodontitis classification

All patients underwent periodontal examination and evolution of panoramic x-rays. The stage and extension of the periodontitis were then classified by the same examiner, with broad experience as a periodontist, based on the International Classification of Periodontal Diseases [2017, 25].

### Dental caries detection

Dental caries examinations were performed by trained and calibrated dental students (Kappa > 0.7), as described elsewhere [23]. Briefly, the presence of caries was observed and recorded by thorough dental examination under artificial light, in a supine position, using clinical mirrors, WHO probes, and tooth isolation with cotton rolls. After tooth cleaning and drying, the visual-tactile inspection was performed to record active and inactive coronal caries lesions, based on the Nyvad criteria [27]. Caries activity (the number of surfaces with either non-cavitated or cavitated caries) and the traditional DMFS (WHO criteria; at the cavity level, representing the past caries experience) were evaluated.

### Salivary pH

The salivary pH was tested on the stimulated saliva using the pH-Fix® indicator strips (Macherey-Nagel GmbH & Co. KG- Düren, Germany). After 1 min of immersion in the saliva, the result was compared to the standard table, as indicated by the manufacturer. The buffering capacity was used for adjustment in the multivariate analysis. It was measured from 1 mL of stimulated saliva; then 3 mL of 0.005 M hydrochloric acid was added, and the pH was measured with an indicator strip after 2 min.

### Salivary DNA extraction and sequencing

DNA was extracted from saliva using the QIAamp DNA Mini Kit (Qiagen), following the manufacturer’s protocol. The V4 region of the *16S rRNA* gene was amplified using the Q5 High Fidelity DNA polymerase kit (New England BioLabs Inc., Life Technologies Inc., MA) and the 564F (TCG-TCG-GCA-GCG-TCA-GAT-GTG-TAT-AAG-AGA-CAG-AYT-GGG-YDT-AAA-GNG) and 806R (GTC-TCG-TGG-GCT-CGG-AGA-TGT-GTA-TAA-GAG-ACA-GTA-CNV-GGG-TAT-CTA-ATC-C) primers (Eurogentec, Belgium). PCR generated amplicons with approximately 242 bp length and products were checked in agarose gel electrophoresis. Amplicons were then purified using MicroCLEAN (Microzone ltd, UK). The Nextera XT kit was used for library preparation and adaptor ligation, followed by clean-up with AMPure Beads (Beckerman Coulter, Inc). Amplicon sizes were assessed with the 2200 Tapestation System, and the QuantiT PicoGreen dsDNA Assay Kit was used to quantify the libraries. Amplicons were then paired-end sequenced on the Illumina MiSeq platform (Illumina, San Diego, CA).

### Bioinformatics and statistics analysis

The amplicon sequence variants (ASVs) were generated through the DADA2 pipeline v.1.12.1 [28] in R version 3.6.1 [29]. Reads were trimmed in 15 nt on left side, and the identified Phi-x sequences were removed. Datasets were filtered allowing a maximum of two expected bases errors per read, N called bases were not permitted, and reads were truncated at 220 nt for the forward and 200 nt for the reverse fragments. Error rates were estimated using a training set of reads and inferred to the whole dataset, and sequences were denoised. Denoised reads were merged, and chimeras identified by method consensus were removed. Qualified sequence variants had an average length of 246 bp and were assigned using the Silva v.138 database [30]. Before performing downstream analysis, ASVs assigned to Eukaryote, Chloroplast, and Mitochondria were removed using the Phyloseq package (version 1.34.0) [31].

The Spearman’s correlation was performed to determine the correlation between the taxa and explanatory variables using the Microbiome R package (version 1.12.0) [32]. The taxa presenting a mean relative abundance higher than 0.001% and significant association (p < 0.01) to the variables tested were plotted in a heatmap. The Canonical Correlation Analysis (CCA) was performed using the vegan R package (version 2.5–7) [33] and plotted using the ggrepel package (version 0.9.1) [34]. The significance of correlation between canonical axes and explanatory matrix was tested with 10,000 permutations.

The alpha diversity was estimated for a dataset of sequence variants rarefied to 35,000 sequences per sample, by the rarecurve function from the vegan R package (version 2.5–7) [33]. The Shannon’s index, Chao1ʹs index and the Pielou index of samples were determined using the Microbiome R package (version 1.12.0) [32], for univariated comparison between groups and bivariate comparisons between groups and pH (alkali – pH 8, neutral – pH 7 or acidic – pH 6) or salivary glucose (≥0.35 or <0.35 mg/dL). The square-root transformed relative abundances of sequence variants combined at the genus level (or the highest taxonomic level annotated) were used to build matrices of similarity based on the Bray-Curtis dissimilarity. The ordination distance was plotted in a non-metric multidimensional scaling (nMDS) using the Phyloseq package (version 1.34.0) [31].

Microbial taxa with differential abundance between groups were identified by DESeq algorithm with the Benjamini-Hockenberg (BH) correction test. Results were obtained using DESeq2 package (version 1.30.1) [35].

Mean and standard deviations were calculated for clinical parameters. The relative abundances at different taxonomic levels were used to evaluate comparisons within and between groups regarding the salivary pH and salivary glucose. Pearson’s correlation, Mann–Whitney and Kruskal–Wallis non-parametric tests were applied for data comparison using SPSS (SPSS Inc. version 26, Chicago, IL).

Network analysis was performed for the modified centered log ratio (mclr) normalized data, using the spring association method from the NetCoMi package (version 1.0.2) [36]. Differences into the taxa association between sample groups were tested with the cluster fast greedy method.

## Results

### Clinical characteristics

Saliva samples were obtained from 52 individuals who underwent dental examinations. Samples from four individuals were excluded from analysis due to missing data. From the remaining sample, 23 individuals had a clinical diagnosis of T2D, from which 10 were insulin users and the remaining used other hyperglycemic medication. The same individuals had periodontitis: n = 6 had stage 4 generalized periodontitis, n = 11 had stage 4 localized periodontitis; n = 6 had stage 3 localized periodontitis. Their HbA1c and FBG levels varied from 6 to 14.2% and 47 to 310 mg/dL, respectively, confirming a great range of glycaemia levels. Twenty-five other individuals were systemically and periodontally healthy, all of them presenting HbA1c lower than 6% and FBG lower than 120 mg/dL. Glycaemic markers, either from saliva or blood, were significantly higher in T2D individuals. However, the local hyperglycaemia varied more, and six subjects had T2D diagnosis but salivary glucose levels were below 0.35 mg/dL, while seven subjects had salivary glucose >0.35 mg/dL and had no diagnosis of T2D (Table 1). Patients with T2D (average age = 58 ± 8) were slightly older than patients with no-T2D (average age = 43 ± 13) (p < 0.001). Besides their hyperglycaemic state, their frequency of 24 h-sucrose intake was higher than for no-T2D, similar to their caries experience (DMFS). This pattern was not observed for active caries (active D-S component). The salivary pH and the salivary glucose had a weak negative correlation (r = −0.3; p = 0.04).

**Table 1.** Clinical characteristics of the samples. No-T2D (systemically and periodontally healthy individuals); T2D (individuals with type 2 diabetes mellitus and periodontitis)

	no-T2D N = 25		T2D N = 23		
	Average	SD	Average	SD	p
Sociodemographic and habits					
Age	43.13	12.98	58.52	8.5	<0.001*
Sex – N(%) female	19 (76%)		35 (72%)		0.748**
24-h sucrose frequency****	0.7	0.9	2.1	1.6	0.005*
Glucose levels					
Salivary glucose (mg/dL)	0.37	0.5	0.84	0.65	0.002*
<0.35 mg/dL (N)	14		6		0.008**
≥0.35 mg/dL (N)	7		17		
HbA1c (%)	5.25	0.41	8.83	2.02	<0.001*
FBG (mg/dL)	89.67	13.07	148.13	65.43	<0.001*
Dental caries					
DMFS (WHO criterium)	36.06	21.01	83.41	31.41	<0.001*
Caries active extent (active D-S)	2.00	3.9	1.14	1.69	0.637*
Other salivary characteristics					
Unstimulated salivary flow – N (%)					
Assialia	3 (12%)		3 (13%)		0.440**
Hypossalivation	10 (40%)		13 (56.5%)		
Ideal	12 (48%)		7 (30.4%)		
Salivary pH	7.08	0.49	7.04	0.64	0.850*
Acidic – pH 6 (N)	2		4		0.483***
Neutral – pH 7 (N)	19		14		
Alkali – pH 8 (N)	4		5		

*U Mann–Whitney test; **Fisher exact test; SD = Standard deviation; WHO = World Health Organization criteria for caries detection; ***Chi-square test; ****Missing data = 6 individuals

### Sequencing output

The dataset from saliva samples sequencing, after screening and optimization, resulted in 2,281 ASVs. Seventy-eight ASVs belonged to the Archaea domain and 2,203 ASVs to the Bacteria domain. Archaea represented 0.01% of the reads, and 33 samples presented at least one taxon belonging to the Archaea domain. The overall salivary microbiota was composed of 33 phyla, 61 classes, 130 orders, 194 families, 332 genera and 407 different taxa annotations. A total of 47 samples were included in downstream analyses after quality checking.

### Correlation of taxa with glycaemic markers and clinical parameters

**Figure 1.**
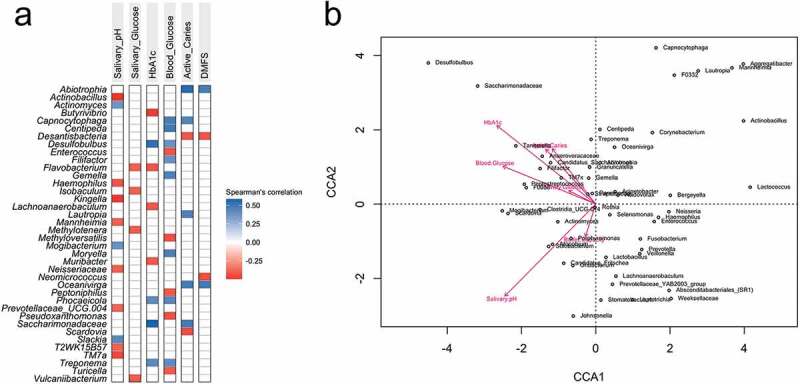
(a) Spearman correlation between taxa with glycaemic markers and clinical variables (p < 0.01). (b) PERMANOVA test plot (number of permutations: 10,000) for CCA under reduced model (53 taxa with abundance higher than 1%, N = 47).

There were 119 taxa significantly correlated (p < 0.05) to at least one of the analysed clinical parameters, including the three glycaemic markers and 38 out of 119 taxa with p-value < 0.01 (see Supplementary Table 1 for correlation values). What stands out from this result is the positive correlation between both blood glucose levels (FBG and HbA1c) with *Treponema, Desulfobulbus* and *Phocaicola*. The salivary pH had the highest number of negatively correlated taxa: *Actinobacillus, Haemophilus, Kingella, Mannheimia, Neisseriaceae, Prevotellaceae* UCG.004, T2WK15B57, and TM7a. *Abiotrophia* and *Oceanivirga* were positively correlated while *Desantisbacteria* was negatively correlated with both caries variables (active caries extent and DMFS, representing caries activity and past caries experience, respectively), with strength of correlations between 0.4 and 0.6 (Figure 1(a)).

The CCA multivariate analysis under-reduced model confirmed statistical significance for salivary pH (p = 0.04) and HbA1c (p = 0.02) (53 taxa with abundance >1%) (Table 2, ANOVA multivariate analysis). Buffer capacity, salivary glucose, FBG and caries (activity and experience) were not significantly affecting the microsystem. Caries indices (active caries extent and DMFS) were concurrent with all blood indexes (HbA1c, FBG and salivary glucose), and *Desulfobulbus* and *Saccharimonadaceae* followed the increase of all those parameters (Figure 1(b)).

**Table 2.** ANOVA CCA permutation test under reduced model for glycaemic markers and clinical parameters

Factor	F value	p – value
**Salivary pH**	**1.6218**	**0.0447**
Buffer capacity	0.4605	0.9776
Salivary glucose	1.1879	0.2518
**HbA1c**	**1.8194**	**0.022**
Fasting blood glucose	0.7376	0.8054
Active caries	0.8324	0.6562
DMFS	0.6739	0.8581

### Diversity and relative abundances of the salivary microbiome

**Figure 2.**
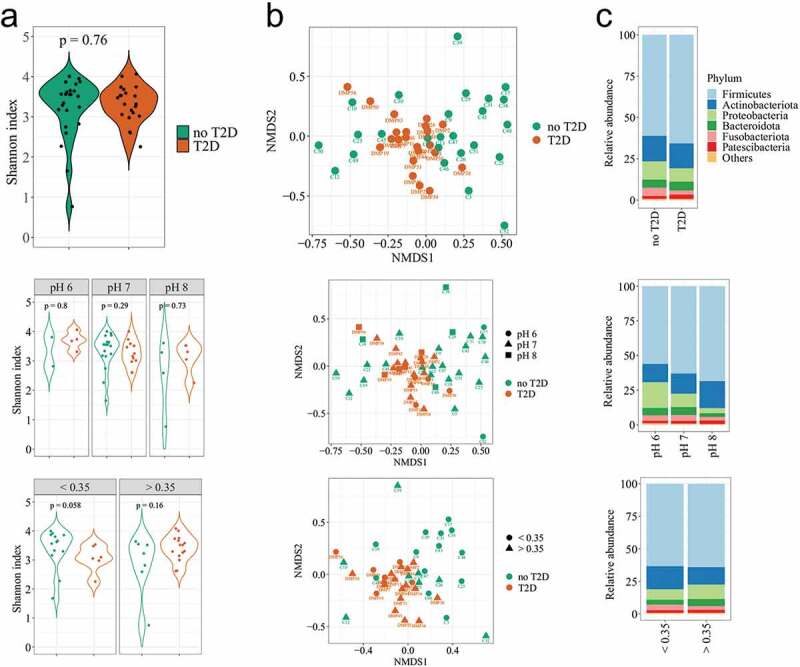
Diversities of the salivary microbiome according to the T2D diagnosis, salivary pH, and salivary glucose (<0.35 mg/dL or >0.35 mg/dL). (a) Alpha-diversity index (Shannon index). B = Beta diversity measured by the Bray-Curtis distance on nMDS plot (n = 390 taxa); C = Most abundant phyla in the salivary microbiome.

The diagnosis of T2D was used to compare diversity and relative abundances, so as the salivary pH that represented the clinical parameter with the highest significance in the CCA multivariate analysis. The salivary glucose was also tested, as the glycaemic marker that varied most independently of the T2D diagnosis, and represented the local hyperglycaemia. There was no difference for alpha-diversity in the salivary microbiome regarding the diagnosis of T2D (Figure 2(a), Supplementary Figure 1, Supplementary Table 2), although both groups presented differences in clinical characteristics that should substantially shape the oral microbiome (age, hyperglycaemia, diagnosis of T2D, periodontitis, caries experience). The alpha-diversity was calculated for samples rarefied at 35,000 reads. Two samples (one with no-T2D and another with T2D) were removed from the set of analysis due to lower counts of reads (Supplementary Figure 2). A borderline result showed higher diversity in individuals without a diagnosis of T2D when they had low salivary glucose (Figure 2(a)).

The compositional dissimilarity among samples was calculated by the PERMANOVA test, for 10,000 permutations. It showed significant variability in community composition regarding the diagnosis of T2D (F = 1.93; p = 0.02), with respect to pH (F = 1.58; p = 0.01), and the salivary glucose (F = 2.16; p = 0.01) (Table 3). In this analysis, the systemic hyperglycaemia was categorised (HbA1c < 6.0%/>6.0%; FBG = <100 mg/dL/>100 mg/dL), so that periodontitis (localised/generalised), showed no significant differences. A cluster of samples was observed regarding the salivary glucose, and the pH 6 was slightly far from the others (Figure 2(b)).

**Table 3.** PERMANOVA multivariate test, showing the influence of the glycaemic markers and clinical variables in the salivary microbiome. T2D = individuals with diagnosis of type 2 diabetes mellitus, no-T2D = individuals without type 2 diabetes mellitus

Factor	Group	F value	p – value
**Diagnosis of T2D**	**T2D/no-T2D**	**1.9265**	**0.0241**
**Salivary pH**	**pH 6/pH 7/pH 8**	**1.5815**	**0.0098**
**Salivary glucose**	**<0.35mg/dL/>0.35mg/dL**	**2.1604**	**0.0097**
HbA1c	<6.0%/>6.0%	0.9949	0.3372
Fasting blood glucose	<100 mg/dL/>100 mg/dL	1.5547	0.0871
Periodontitis extent	Localised/Generalised	1.2732	0.1874

Twenty-three out of 33 phyla presented very low abundance and prevalence. The average number of phyla was 22 in T2D and 25 in no-T2D samples. Regarding the archaeal content, the *Euryarchaeota* phylum (which includes methanogenic organisms) was detected in only two samples, while *Halobacterota, Chrenarchaeota* and *Nanoarchaeota* were more prevalent archaeal phyla, but representing no more than 0.001% of the total reads (Supplementary Figure 3). The 10 most abundant phyla presented no differences in their relative abundance regarding the diagnosis of T2D (Figure 2(c)). The increase of the salivary pH was followed by a clear reduction of *Proteobacteria* and an increase in *Firmicutes* and *Actinobacteriota*. The relative abundance of *Actinobacteriota* was also affected by the salivary glucose (Figure 2(c)), which was confirmed when the taxa average differences were compared. The *Actinobacteriota* phylum showed higher abundance in the group of individuals with salivary glucose <0.35 mg/dL than in the group with salivary glucose ≥0.35 mg/dL (17.6 ± 6.8% vs. 13.1 ± 5.1%; p = 0.01))(Table 4, p = 0.08). The *Firmicutes/Bacteroidota* ratio significantly increased with the alkalinisation of the salivary pH (p = 0.03). The opposite was observed for *Proteobacteria* that seemed to be increased in abundance through saliva acidification (pH 6; p = 0.003). *Bacteroidota* (p = 0.02), and*Spirochaetota* (p = 0.01) were in low abundance, butwere most likely affected by the salivary pH. Regarding the genus level, it is worth to mention that *Veillonella* was enriched in the acidic saliva (p = 0.01). These estimations were performed based on non-parametric calculations, considering the small size of the saliva samples at acidic and alkali pHs (Table 4).

**Table 4.** Relative abundance (%) of salivary microbiome taxa significantly influenced by the salivary pH and/or salivary glucose

	Acidic salivary pH (n = 6)	Neutral salivary pH (n = 32)	Alkali salivary pH (n = 9)	p*	Salivary glucose < 0.35 mg/dL (n = 21)	Salivary glucose ≥ 0.35 mg/dL (n = 26)	p
Rate *Firmicutes/ Bacteriodota*	16.83 ± 10.12	25.06 ± 32.04	81.88 ± 120.41	0.03	49.94 ± 89.29	23.89 ± 26.16	> 0.05
*Proteobacteria*	0.16 ± 0.12	0.11 ± 0.10	0.04 ± 0.04	0.003	0.09 ± 0.07	0.12 ± 0.12	> 0.05
*Actinobacteriota*	0.13 ± 0.04	0.15 ± 0.05	0.20 ± 0.09	0.08	0.18 ± 0.07	0.13 ± 0.05	0.01
*Bacteroidota*	0.05 ± 0.03	0.06 ± 0.04	0.02 ± 0.02	0.02	0.04 ± 0.03	0.05 ± 0.04	> 0.05
*Spirochaetota*	0.01 ± 0.01	0.00 ± 0	0	0.01	0.001 ± 0.002	0.002 ± 0.004	> 0.05
*Veillonella* spp.	0.03 ± 0.02	0.04 ± 0.03	0.01 ± 0.01	0.01	0.03 ± 0.02	0.03 ± 0.03	>0.05

**Figure 3.**
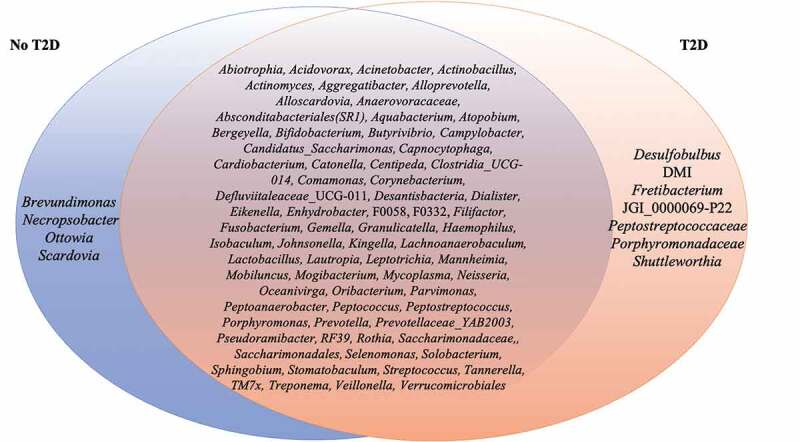
List of members of the ubiquitous microbiome (present in at least 50% of the samples). Blue represents no-T2D, while Orange represents T2D. The intersection represents the ubiquitous taxa present in all samples.

Fifty-three genera had abundance higher than 1% (Supplementary Figure 4). Several organisms in abundance lower than 0.01% were detected in at least 50% of the samples, such as *Aggregatibacter* (m = 0.001%, 0–0.09%), *Bifidobacterium* (m = 0.0001%, 0–0.012%), *Capnocytophaga* (m = 0.002%, 0–0.064%), *Lactobacillus* (m = 0.0001%, 0–0.013%), *Verrucomicrobiales* (m = 0.0002%, 0–0.008%), amongst others (Figure 3). Some of these microorganisms were ubiquitous taxa in individuals with the diagnosis T2D, such as *Desulfobulbus* (m = 0.0002%, 0–0.002%). Others, such as *Brevundimonas* (m = 0.0001%, 0–0.0008%) were ubiquitous taxa in no-T2D samples.

**Figure 4.**
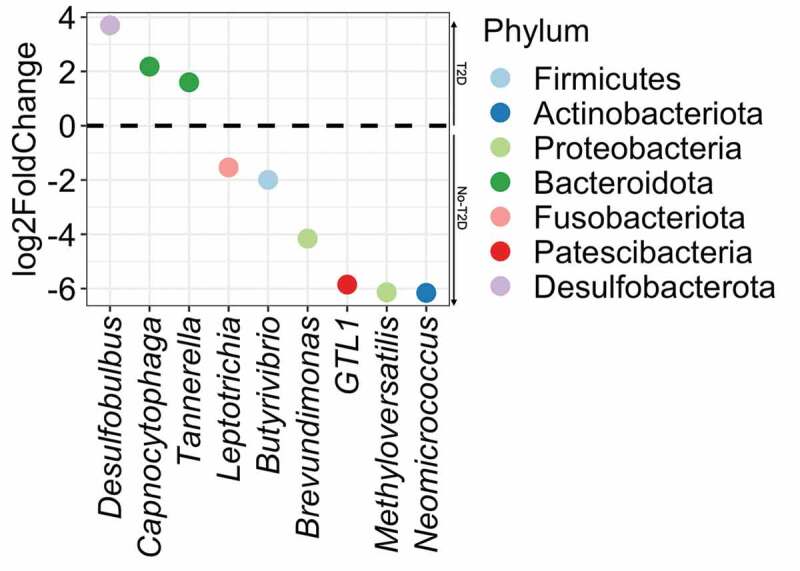
Differential abundance plot calculated by DESeq2. Taxa with positive log2 fold-change values are significantly more abundant (enriched) in T2D and negative log2 fold-change values are enriched in no-T2D.

Deseq2 analysis showed significant differential abundance for nine taxa (p < 0.05; BH correction) out of 390 taxa (Figure 4). *Desulfobulbus*, described above as being ubiquitous in T2D samples, correspondingly had the greatest enrichment in individuals with T2D (*Desulfobaterota* phylum, log2FC = 4), followed by *Bacteroidota* (*Capnocytophaga* and *Tannerella* genus, log2FC = 2). *Actinobacteriota*, (*Neomicrococcus* genera, log2FC = −6) and *Proteobacteria* (the *Methyloversatillis* and *Brevundimonas* genus) were the most significantly enriched organisms in T2D samples. *Patescibacteria* (*Saccharimonadaceae* GTL1 and *Butyvibrio*), as well as *Fusobacteriota* (*Leptotrichia*) were also significantly more abundant in the samples from individuals diagnosed with T2D.

**Figure 5.**
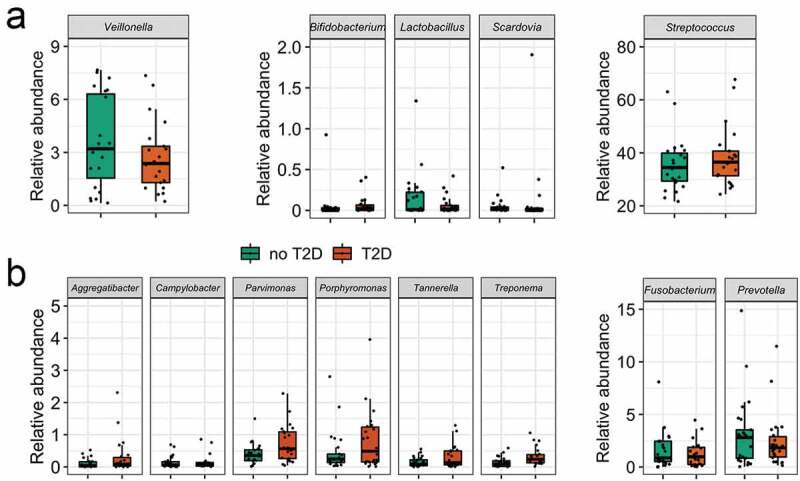
Relative abundance (%) in T2D and no-T2D groups of (a) acidogenic caries-associated taxa, and (b) proteolytic periodontitis-associated taxa. Wilcoxon test (significance level <0.05).

As a complementary analysis, the average abundances of deliberately selected genera (usually linked to dental caries) were compared to test the hypothesis of the enrichment of typical acidogenic/acidophilic taxa due to the hyperglycaemia state. No differences were observed for the five acid-related genera in T2D and no-T2D samples (from 390 taxa from 47 samples) (Figure 5(a), Supplementary Table 3). Interestingly, the same was observed for the proteolytic pathobiont taxa, except for *Treponema*, that was significantly more abundant in T2D samples (Figure 5(b)).

### Network analysis

**Figure 6.**
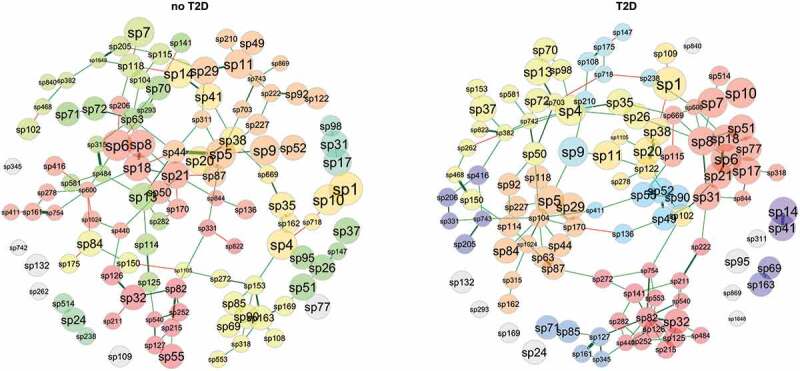
Network analysis (clusters are differentiated by colours; node size based on mclr values). Green edges correspond to positive estimative association and red edges correspond to negative association between taxa. Similarity between clusterings – Adjusted Rand index = 0.038 (p-value = 0.007).

The microbial network profiles of samples from individuals diagnosed with T2D and no-T2D considered 110 taxa that were prevalent in at least 10 out of 47 samples (Figure 6). Distance of centralities (degree, betweenness, eigenvector, and closeness) were tested using the Jaccard index. Degree (p < 0.05), betweenness (p < 0.01), and eigenvector (p < 0.001), but not for closeness (p > 0.1) presented significant differences between groups (Supplementary Table 4). Keystone taxa were not identified by the centrality values.

The node pairs were connected by the shortest path in T2D samples compared to those in no-T2D, indicating that the T2D microbiota taxa had better interconnected clusters. This can be particularly relevant for taxa nodes associated with *Acidovorax* (sp82) and *Acinetobacter* (sp32) (dark red cluster in T2D) or for nodes associated with *Actinomyces* (sp6), *Granulicatella* (sp7) and *Solobacterium* (sp8) (red cluster in T2D) and for nodes associated with *Eikenella* (sp104) and *Haemophilus* (sp5) (orange cluster in T2D) (see Supplementary Table 5 for the network taxa ID annotation).

*Methyloversatilis* (sp345), significantly enriched in the no-T2D samples, clustered with *Saccharimonadaceae* (sp71), *Actinomycetaceae* F0332 (sp85), *Ottowia* (sp161), and *Aquabacterium* (sp127) (blue cluster in T2D). An interdomain network was observed between archaea-bacterial taxa. *Aenigmarchaeum* (sp840) and *Woesearchaeales* SCGC_AAA286-E23 (sp869) comprised unconnected taxa (grey nodes) in the T2D, however in no-T2D were positive and directly associated with *Saccharimonadaceae* GTL1 (sp382), and a *Saccharimonadales* species (sp95), respectively.

Regarding the taxa with significant differential abundance, *Desulfobulbus* (sp147) stood out in the network analysis. It was within a cluster of no-T2D samples, in positive association with *Peptostreptococcus* (sp26), *Saccharimonadales* species (sp95), *Parvimonas* (sp37) and *Anaerovoracaceae* species (sp51) (green cluster). In the T2D, *Veillonella* (sp9), *Bifidobacteria* (sp175), and *Scardovia* (sp108), amongst others (light blue cluster) are clustered in direct or indirect negative association with *Desulfobulbus*. This indicates an important opposition to the caries-associated and butyrate-producing bacteria.

A central role of *Veillonella* (sp9) confirmed its importance in the T2D microbiome, when combined with the previously described higher relative abundances in acidic than alkali pHs. *Veillonella* appears as a connector node linking three clusters in positive association with *Dialister* (sp210) (light blue cluster), with *Haemophilus* (sp5) (orange cluster) and with *Prevotella* (sp11) (gold cluster). In the no-T2D samples, we cannot see *Veillonella* as a connector node taxon, but it is clustered with direct or indirect positive association with *Haemophilus* (sp5), *Dialister* (sp210) and *Prevotella* (sp11). It suggests *Veillonella* as a multifunctional taxon capable of maintaining the bacterial network structure of the oral microbiota in T2D samples.

*Butyrivibrio* (sp102) was differentially more abundant in no-T2D samples and also seemed to have a role as cluster connector within the T2D samples. In no-T2D, it was negatively linked to the *Anaerovoracacea* family XIII UCG-001 (sp468), while in T2D it comprised the same cluster of *Prevotella* (gold cluster) and positively associated with *Atopobium* (sp21) (red cluster), *Novosphingobium* (sp754) (dark red cluster), and *Catonella* (sp122) (gold cluster). Another differentially abundant bacterium *Leptotrichia* (sp20) was enriched in no-T2D, and in the network analysis was positively and directly associated with *Stomatobaculum* (sp38) in both groups. Differentially abundant bacteria in T2D, *Tannerella* (sp90), was positively associated with *Johnsonella* (sp55) and clustered with *Selenomonas* (sp52), *Prevotellaceae* YAB2003 group (sp49), *Alloscardovia* (sp136) and *Peptostreptococcales-Tissierellales* W5053 (sp411) (blue cluster). *Tannerella* is known as an abundant component in periodontitis sites, and it may reflect its importance as a link in the network of T2D, where most individuals had stages 3/4 localised periodontitis.

Pondering the strongest positive links in the T2D, some correlations may be highlighted: *Pseudoramibacter* (sp222) with *Parascardovia* (sp211) and *Bifidobacterium* (sp175) with *Scardovia* (sp108). *Pseudoramibacter* represents a very common microorganism in root canals, while all others belong to the family *Bifidobacteriaceae*. Meanwhile, strong positive association in no-T2D samples included the fermenter and nitrate-reducer *Actinobacillus* (sp44) and *Haemophilus* (sp5), a bacterial genus found in all oral cavity sites of patients. Also, in no-T2D samples, there was a positive association of *Desulfobulbus* (sp147) and the infection-associated *Peptostreptococcus* (sp26).

## Discussion

Some oral microbial signatures such as *Desulfobulbus* were associated with all hyperglycaemia indices tested. Members of this genus were favoured by the hyperglycaemia state showing significant positive correlation with FBG and HbA1c levels. *Desulfobulbus* was ubiquitous and highly abundant in individuals with T2D. Moreover, *Desulfobulbus* seemed to be an antagonist to caries-associated and butyrate-producing bacteria. The increase in blood glucose levels was significantly linked to the increase in *Treponema, Phocaiecola* and *Saccharimonadaceae* species. On the other hand, *Actinobacteriota* may be depleted in the hyperglycaemia state, as taxa belonging to this phylum were highly abundant in no-T2D samples and in individuals with salivary glucose <0.35 mg/dL. A notable influence of the salivary pH on the oral microbiome was observed through changes in the proportions of the *Firmicutes/Bacteriodota* ratio (increasing in the saliva with alkali pH). In addition, we found the highest number of negatively correlated taxa between all the explanatory variables (Figure 1(a), showing eight taxa that were significantly negatively correlated with the salivary pH).

Our observations are consistent with the broader hypothesis that microbial communities along the digestive tract might be risk factors for diabetic regulation in diabetic individuals, such as the increased abundance of sulphate-producers in the hyperglycaemic state. The microbiota may also be involved in the early onset of diabetes development [37]. It was shown elsewhere that the dysbiosis index explained 6% of the variation in longitudinal glucose change, predicting 2 year glucose change among diabetes-free individuals [38]. Likewise, the glycaemic control led to a shift in the oral microbial population resembling that of healthy individuals, which are complex and biodiverse [19]. In this context, the salivary microbiome of individuals with T2D had higher abundance of *Desulfovibrionaceae*, a taxonomic order associated with dyslipidaemia, obesity [39] and DM [40]. This is a sulphate-reducer that can perform anaerobic respiration utilizing sulphate as a terminal electron acceptor. Sulphate-reducing bacteria are the main H_2_S generators in the gut microbiome, with a potential role in the individual’s metabolic condition and complication related to DM. A relationship between H_2_S and gut microbial dysbiosis signalling and function has been suggested [41,42], as increased H_2_S levels disturb the pancreatic β-cell function and decrease insulin secretion [41]. Furthermore, *Desulfovibrio desulfuricans* are trimethylamine oxide generators, which is similarly correlated with the risk of metabolic syndromes [41]. Another sulphate-reducer was found to be a protagonist in this study, *Desulfobulbus*, which has previously been referred to as a periodontal pathobiont, because it induces proinflammatory response and secretes potential protein toxins [43]. Although in low abundance, it was significantly enriched in subgingival sites with periodontitis [44]. Therefore, the presence of this sulfidogenic microorganism in hyperglycaemic and periodontitis microbiomes is easily understandable. Systemically, a significantly higher abundance of sulphate-reducing bacteria in the oral microbiome can indicate saliva as a potential biomarker of DM-dysbiotic gut microbiome.

Gut and oral diabetic microbiomes may be more connected than we expected. We found a significantly negative association between *Desulfobulbus* and butyrate-producing bacteria from the *Bifidobacteriaceae* family in the diabetic oral microbiota. Locally, members of the *Bifidobacteriaceae* family are strongly associated with caries as their fermentation end products are mainly organic acids. These metabolic products can reduce the pH, leading to critical acidity levels for tooth demineralisation, indicating a potential link to the increased prevalence of caries in diabetic individuals [7]. Organic acids can be converted into short-chain fatty acids (SCFAs) by butyrate-producing bacteria through cross-feeding interactions [45]. Systemically, the SCFAs including butyrate serve as key mediators of microbial-host signalling and are linked to a better insulin response [46]. For instance, *Bifidobacterium* spp. have anti-inflammatory properties and protect the epithelial barrier by reducing lipopolysaccharides and the trimethylamine N-oxide (TMAO) influx into the blood [21]. Our results on the salivary microbiota are in line with the decreased butyrate-producing bacteria levels in the gut microbiome of DM individuals [47]. This is the case of the butyrate-producer *Butyrivibrio*, belonging to the *Clostridiales* order, that was significantly depleted in the T2D samples and presented a central role in the network analysis. Changes in the SCFAs metabolism in the diabetic gut microbiome are linked to the enrichment of *Bacteriodota*, which was also found to be enriched in our data. A biomarker of this event is the *Firmicutes/Bacteriodota* ratio. It has been positively linked with blood glucose levels [46], and we showed its significant reduction in the salivary microbiota among individuals with lower salivary pHs.

Other oral phyla were reduced in the hyperglycaemia state. *Actinobacteriota* was significantly lower in salivary glucose >0.35 mg/dL, while *Proteobacteria* (*Methyloversatillis* and *Brevundimonas* genus) were the most significantly depleted organisms in T2D samples. Many pathobionts are members of the *Proteobacteria* phylum, and a high proportion of such organisms may have a pro-inflammatory effect in diabetic subjects. This observed imbalance in the microbial composition can be a result of the gut dysbiosis and potentially impair the insulin resistance. *Leptotrichia* was likewise associated with samples without T2D and is a representative of the core microbiome, present in almost all individuals [48,49], as a bridge between early and late colonizers within oral biofilms. New studies investigating microbial functions are necessary to explain connections with *Stomatobaculum*, as observed in our network analysis, independently of the sample group status.

The altered blood sugar status can disrupt homeostasis, providing a more profound change on the microbiota profile particularly when combined with periodontitis [19,50]. It is essential to point out that genera strongly associated with periodontitis, *Tannerella* and *Treponema*, demonstrated a connection with increased pH, the diagnosis of T2D, and the blood glycaemic levels. TM7, *Neisseriaceae* [G-1] bacterium HMT-174 (F0058) and *Tannerella* demonstrated a positive correspondence with HbA1c, FBG, and salivary glucose in the CCA multivariate analysis. Additionally, *Tannerella* had several links in the T2D-associated microbial network. Since all diabetic individuals included in the present study were also diagnosed with some level of periodontitis, it was not possible to clarify if the higher level of some periodontal-related taxa was influenced by the T2D condition or by periodontitis, although the periodontitis extent was included in the PERMANOVA multivariate test, showing no significant impact in the analysis. Despite this potential limitation, it is important to highlight both the results of the Spearman correlation, as well as of the canonical correlation analysis, demonstrating that all the glucose parameters profoundly impacted the salivary microbiota changes. Hence, it is the glycaemic status rather than the T2D diagnosis that perhaps should be considered a biomarker related to salivary dysbioses.

Another obvious factor influencing the oral ecosystem of individuals with T2D was the significant salivary dysfunctions, such as pH changes. Goodson et al. evaluated changes in abundance of some bacterial species in the saliva of adolescents with high concentrations of salivary glucose, showing that the higher the salivary glucose, the lower the pH of the saliva [8], which we confirmed here. As glucose is a well-known energy source for many oral bacteria, changes in its concentration would lead to reduced overall bacterial diversity, favouring acidic and acidogenic bacterial species. We showed an enrichment of the *Abiotrophia* and *Oceanivirga* in the oral microbiota with the increase of active caries extent and DMFS. For instance, *Oceanivirga* has been found in pharyngeal infections [51], while *Abiotrophia* was enriched in adolescents from a community with high caries prevalence when compared to the ones from a low caries prevalence community [52]. Meanwhile, *Desantisbacteria* was negatively correlated with both caries variables and significantly affected by the salivary pH. Indeed, the salivary pH had the highest number of negatively correlated taxa and significantly changed the beta diversity and the CCA multivariable analysis. This confirmed the relevance of the pH changes in the diabetic microbiome, even though the results did not directly change the proportion of the typical acidogenic microbiota regarding the diabetes status. Furthermore, there was a central role of *Veillonella* spp. in the bacterial network of the diabetic salivary microbiome (Figure 6), and they were significantly enriched in the acidic saliva (Table 4). Members of this genus are linked to the classical Socransky’s purple-complex [53], and their lactate metabolism might facilitate the pH neutralization in biofilms [54]. They have been already related to hyperglycaemia elsewhere [55] and dental caries [56]. These characteristics might explain the significant higher proportions of these organisms in lower pH environments. Furthermore, they are health-associated organisms in periodontal sites [57].

Our results confirmed the importance of analysing not only the main taxa present but also the microorganisms in low abundance, as these may be impacted by clinical parameters. Current research on the salivary microbiome has mainly been restricted to the identification of the most abundant microbiota associated with health or disease. We believe that this strategy could cause an incomplete misunderstanding of the ecology and environment as metabolic functions exerted by low-abundant microorganisms can be linked to the dysbiotic microhabitats in a sort of ‘butterfly effect’ [58]. This can be clearly observed by the inclusion of *Desulfobulbus* in the analysis, even at very low relative abundance. Although representing a minority taxon, its ubiquity and association with clinical parameters were found to be consistent. Besides, the network analysis indicated its important role in the microbiome, as discussed above.

The analysis comparing the diagnosis of T2D vs. no-T2D should be interpreted with caution, considering confounding aspects affecting all differences between samples. Those factors represent additional selective pressure over the microbiota composition in the diabetic group. Also, in general, the dichotomization of individuals did not separate the ones with controlled from uncontrolled glucose levels, those with long-term diagnostic of DM and use of hypoglycaemic drugs were not taken into account either. To overcome this issue, we performed several analyses without considering the diagnostic of T2D, instead taking into account the glycaemic status using HbA1c, FBG and salivary glucose as continuous variables. Salivary glucose showed some influence in the salivary microbiome, and this trend should be further studied using a more sensitive test for salivary glucose measurement. Although it is not possible to confirm that salivary glucose plays an important role in disturbing the microsystem, perhaps it favours microorganisms that influence the pH balance. In this case, the salivary pH would be indirectly influenced by the salivary hyperglycaemia, although it is not the microbiota typically acidogenic that is enriched in the hyperglycaemic state. Other clinical parameters, not evaluated here, could also be involved in the imbalance of the diabetic microbiome, such as smoking and adiposity. Future perspectives in this field include the development of a longitudinal study to confirm these associations, and hence the potential of targeting the oral microbiome as an approach to detect and treat T2D.

In conclusion, the salivary microbiome was shaped by systemic hyperglycaemia, as well as changes in the salivary pH, which may be linked to local hyperglycaemia. Locally, these changes might be related to the oral manifestations of T2D, including their higher caries experience. Systemically, the enrichment of predictive biomarkers of gut dysbiosis in the salivary microbiome can reflect its capacity of impairment of the hyperglycaemia. More than leading to local changes in the oral cavity, the oral microbiome may harbour important biomarkers for the early diagnosis of T2D due to the enrichment of sulphate-reducers and depletion of butyrate-producers. In the context of the integrated hypothesis of caries and periodontal diseases [13], due to the link of sugar-driven hyperglycaemia and inflammation in periodontal tissues, there is a potential to control caries and periodontal diseases by stabilization of blood sugar levels.

## Supplementary Material

Supplemental Material

Supplemental Material

## Data Availability

The sequences were deposited at the National Biotechnology Information Center (NCBI) in BioProject PRJNA807496 (http://www.ncbi.nlm.nih.gov/bioproject/807496).
