# Supplementary material for: Enrichment of sulphate-reducers and depletion of butyrate-producers may be hyperglycaemia signatures in the diabetic oral microbiome
Source: J Oral Microbiol. 2022 Jun 3;14(1):2082727. doi: 10.1080/20002297.2022.2082727 (PMC9176348; doi:10.1080/20002297.2022.2082727)
Supplement: Supplemental Material [file ZJOM_A_2082727_SM8843.docx]

**Supplementary Table 1.** Spearman correlation statistics comparing taxa with glycaemic markers and clinical parameters.

| Taxa | Salivary pH | Salivary Glucose | HbA1c | Fasting Blood Glucose | Active Caries | DMFS |
| --- | --- | --- | --- | --- | --- | --- |
| *Abiotrophia* | ns | ns | ns | ns | 0.551373043 | 0.472223743 |
| *Actinobacillus* | -0.49584019 | ns | ns | ns | ns | ns |
| *Actinomyces* | 0.372459253 | ns | ns | ns | ns | ns |
| *Butyrivibrio* | ns | ns | -0.476575836 | ns | ns | ns |
| *Capnocytophaga* | ns | ns | ns | 0.471197144 | 0.44321523 | ns |
| *Centipeda* | ns | ns | ns | 0.493063446 | ns | ns |
| *Desantisbacteria* | ns | ns | ns | ns | -0.41820966 | -0.42448309 |
| *Desulfobulbus* | ns | ns | 0.53264328 | 0.389084503 | ns | ns |
| *Enterococcus* | ns | ns | ns | -0.426908516 | ns | ns |
| *Filifactor* | ns | ns | ns | 0.395493902 | ns | ns |
| *Flavobacterium* | ns | -0.393415511 | -0.424635141 | ns | ns | ns |
| *Gemella* | ns | ns | ns | 0.458958257 | ns | ns |
| *Haemophilus* | -0.42387903 | ns | ns | ns | ns | ns |
| *Isobaculum* | ns | -0.392470722 | ns | ns | ns | ns |
| *Kingella* | -0.48947809 | ns | ns | ns | ns | ns |
| *Lachnoanaerobaculum* | ns | ns | -0.414670168 | ns | ns | ns |
| *Lautropia* | ns | ns | ns | ns | 0.453101422 | ns |
| *Mannheimia* | -0.38788982 | ns | ns | ns | ns | ns |
| *Methylotenera* | ns | -0.404906864 | ns | ns | ns | ns |
| *Methyloversatilis* | ns | ns | ns | -0.439586024 | ns | ns |
| *Mogibacterium* | 0.390537067 | ns | ns | ns | ns | ns |
| *Moryella* | ns | ns | ns | 0.458165945 | ns | ns |
| *Muribacter* | ns | ns | -0.413391964 | ns | ns | ns |
| *Neisseriaceae* | -0.39840188 | ns | ns | ns | ns | ns |
| *Neomicrococcus* | ns | ns | ns | ns | ns | -0.43779350 |
| *Oceanivirga* | ns | ns | ns | ns | 0.482105682 | 0.486678381 |
| *Peptoniphilus* | ns | ns | ns | -0.41374936 | ns | ns |
| *Phocaeicola* | ns | ns | 0.415106742 | 0.434036513 | ns | ns |
| *Prevotellaceae*_UCG.004 | -0.38753374 | ns | ns | ns | ns | ns |
| *Pseudoxanthomonas* | ns | ns | ns | -0.43213671 | ns | ns |
| *Saccharimonadaceae* | ns | ns | 0.583979497 | ns | 0.485615972 | ns |
| *Scardovia* | ns | ns | ns | ns | -0.44606337 | ns |
| *Slackia* | 0.424884414 | ns | ns | ns | ns | ns |
| T2WK15B57 | -0.38753374 | ns | ns | ns | ns | ns |
| TM7a | -0.45305499 | ns | ns | ns | ns | ns |
| *Treponema* | ns | ns | 0.427051849 | 0.414060197 | ns | ns |
| *Turicella* | ns | ns | ns | -0.41462995 | ns | ns |
| *Vulcaniibacterium* | ns | -0.40776527 | ns | ns | ns | ns |

**Supplementary Figure 1.** Alpha-diversity indexes displayed by diagnosis of T2D, salivary pH, and salivary glucose (< 0.35 mg/dL or > 0.35 mg/dL). **A =** comparison between groups; **B =** comparison between groups and related to the salivary pH; **C =** comparison between groups and related to the salivary glucose.

**
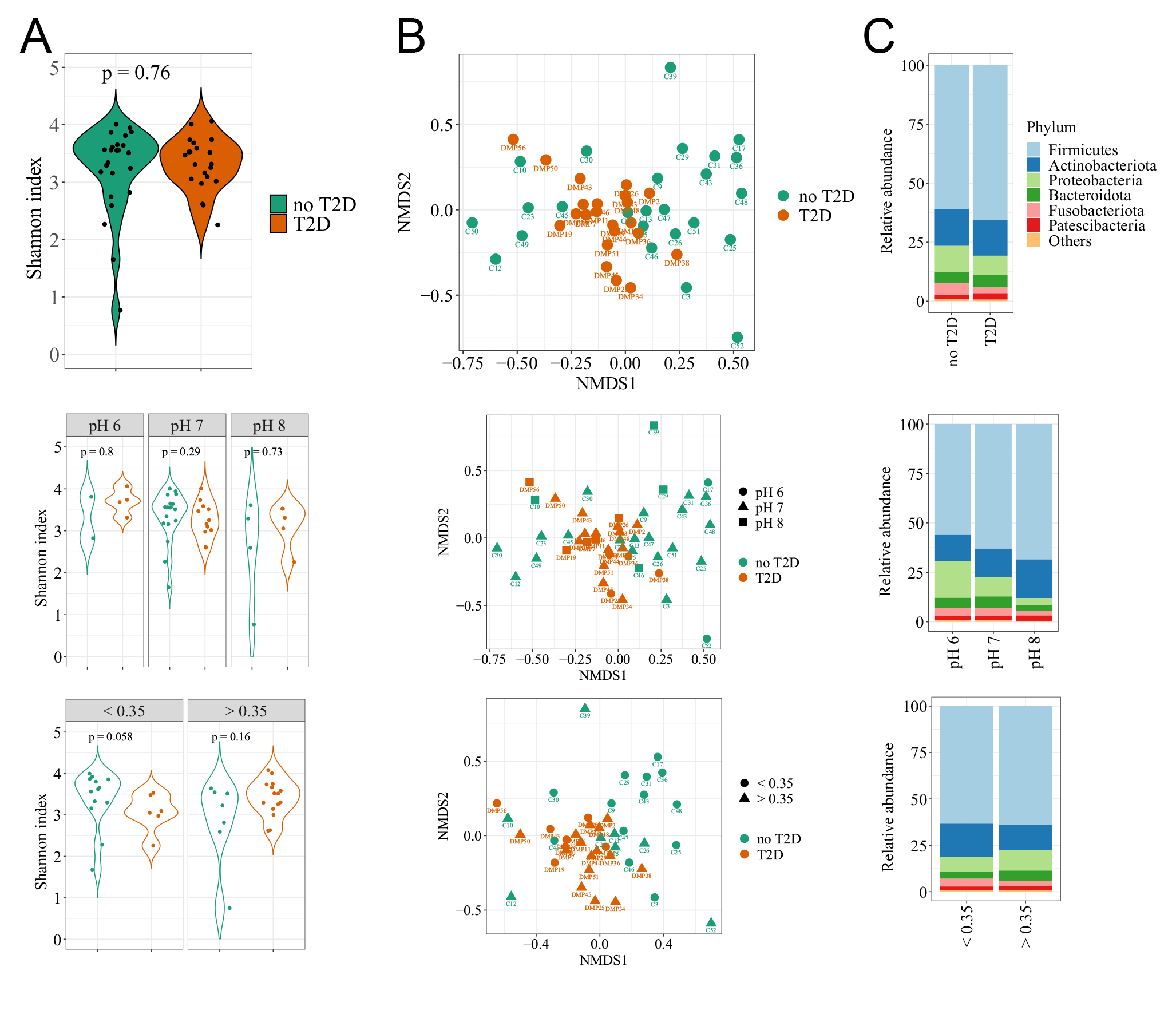

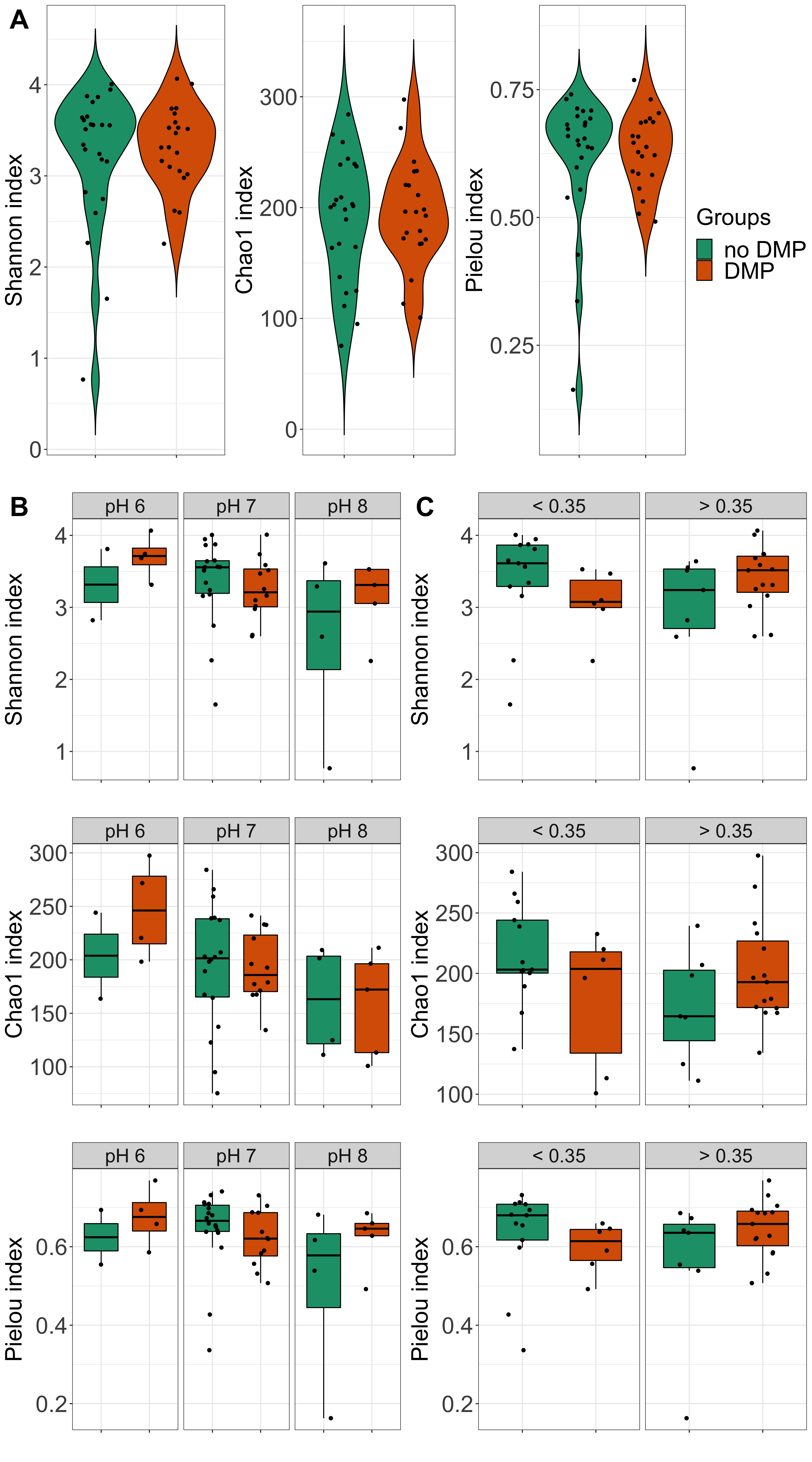
**

**Supplementary Table 2.** Alpha-diversity difference between groups with and without a diagnosis of T2D, salivary pH, and salivary glucose.

| Diversity index | Factor | p - value |
| --- | --- | --- |
| Shannon | All patients | 0.760 |
|  | pH 6 | 0.800 |
|  | pH 7 | 0.290 |
|  | pH 8 | 0.730 |
|  | < 0.35 | 0.058 |
|  | > 0.35 | 0.160 |
| Chao1 | All patients | 0.950 |
|  | pH 6 | 0.530 |
|  | pH 7 | 0.660 |
|  | pH 8 | 0.900 |
|  | <0.35 | 0.320 |
|  | >0.35 | 0.190 |
| Pielou | All patients | 0.570 |
|  | pH 6 | 0.530 |
|  | pH 7 | 0.150 |
|  | pH 8 | 0.410 |
|  | < 0.35 | 0.110 |
|  | > 0.35 | 0.240 |

**Supplementary Figure 2**. Curve before and after rarefaction.

**Supplementary Figure 3.** Archaeal phylum proportion

**Supplementary Figure 4.** Heatmap representing n = 53 taxa with abundance higher than 1% (of 390 taxa from 47 samples) (DMP-labeled samples= T2D; C-labeled samples = no-T2D).





**Supplementary Table 3.** Taxa abundance comparison between individuals with and without a diagnosis of T2D (Wilcoxon test).

| Taxa | p - value |
| --- | --- |
| *Bifidobacterium* | 0.150 |
| *Lactobacillus* | 0.600 |
| *Scardovia* | 0.200 |
| *Veillonella* | 0.270 |
| *Streptococcus* | 0.590 |
| *Aggregatibacterium* | 0.190 |
| *Campylobacter* | 0.870 |
| *Parvimonas* | 0.075 |
| *Porphyromonas* | 0.210 |
| *Tannerella* | 0.180 |
| *Treponema** | 0.004 |
| *Fusobacterium* | 0.530 |
| *Prevotella* | 0.550 |

**Supplementary Table 4.** Jaccard index values for individuals with and without a diagnosis of T2D.

|  | Jaccard (*j*) | P(J ≤ *j*) | P(J ≥ *j*) |
| --- | --- | --- | --- |
| Degree* | 0.175 | 0.02110 | 0.992069 |
| Betweenness centr.** | 0.114 | 0.00292 | 0.999323 |
| Closeness centr. | 0.100 | 0.10404 | 0.982658 |
| Eigenvector centr.*** | 0.120 | 0.00052 | 0.999869 |
| Hub taxa | 0.000 | 1.00000 | 1.000000 |

Significance codes: ∗∗∗: 0.001, ∗∗: 0.01, ∗⁠: 0.05, ..⁠: 0.1

**Supplementary Table 5.** Network taxa ID annotation.

| ID |  |  |  | Taxonomy |  |  |  |
| --- | --- | --- | --- | --- | --- | --- | --- |
|  | Kingdom | Phylum | Class | Order | Family | Genus | Annotation |
| sp1 | Bacteria | Firmicutes | Bacilli | Lactobacillales | Streptococcaceae | Streptococcus | Streptococcus |
| sp4 | Bacteria | Firmicutes | Bacilli | Staphylococcales | Gemellaceae | Gemella | Gemella |
| sp5 | Bacteria | Proteobacteria | Gammaproteobacteria | Pasteurellales | Pasteurellaceae | Haemophilus | Haemophilus |
| sp6 | Bacteria | Actinobacteriota | Actinobacteria | Actinomycetales | Actinomycetaceae | Actinomyces | Actinomyces |
| sp7 | Bacteria | Firmicutes | Bacilli | Lactobacillales | Carnobacteriaceae | Granulicatella | Granulicatella |
| sp8 | Bacteria | Firmicutes | Bacilli | Erysipelotrichales | Erysipelotrichaceae | Solobacterium | Solobacterium |
| sp9 | Bacteria | Firmicutes | Negativicutes | Veillonellales-Selenomonadales | Veillonellaceae | Veillonella | Veillonella |
| sp10 | Bacteria | Actinobacteriota | Actinobacteria | Micrococcales | Micrococcaceae | Rothia | Rothia |
| sp11 | Bacteria | Bacteroidota | Bacteroidia | Bacteroidales | Prevotellaceae | Prevotella | Prevotella |
| sp13 | Bacteria | Proteobacteria | Gammaproteobacteria | Burkholderiales | Neisseriaceae | Neisseria | Neisseria |
| sp14 | Bacteria | Firmicutes | Clostridia | Lachnospirales | Lachnospiraceae | Oribacterium | Oribacterium |
| sp17 | Bacteria | Patescibacteria | Saccharimonadia | Saccharimonadales | Saccharimonadaceae | TM7x | TM7x |
| sp18 | Bacteria | Firmicutes | Clostridia | Peptostreptococcales-Tissierellales | Anaerovoracaceae | Mogibacterium | Mogibacterium |
| sp20 | Bacteria | Fusobacteriota | Fusobacteriia | Fusobacteriales | Leptotrichiaceae | Leptotrichia | Leptotrichia |
| sp21 | Bacteria | Actinobacteriota | Coriobacteriia | Coriobacteriales | Atopobiaceae | Atopobium | Atopobium |
| sp24 | Bacteria | Firmicutes | Bacilli | Lactobacillales | Aerococcaceae | Abiotrophia | Abiotrophia |
| sp26 | Bacteria | Firmicutes | Clostridia | Peptostreptococcales-Tissierellales | Peptostreptococcaceae | Peptostreptococcus | Peptostreptococcus |
| sp29 | Bacteria | Fusobacteriota | Fusobacteriia | Fusobacteriales | Fusobacteriaceae | Fusobacterium | Fusobacterium |
| sp31 | Bacteria | Firmicutes | Clostridia | Clostridia UCG-014 | NA | NA | Clostridia UCG-014 |
| sp32 | Bacteria | Proteobacteria | Gammaproteobacteria | Pseudomonadales | Moraxellaceae | Acinetobacter | Acinetobacter |
| sp35 | Bacteria | Bacteroidota | Bacteroidia | Bacteroidales | Porphyromonadaceae | Porphyromonas | Porphyromonas |
| sp37 | Bacteria | Firmicutes | Clostridia | Peptostreptococcales-Tissierellales | Peptostreptococcales-Tissierellales fa | Parvimonas | Parvimonas |
| sp38 | Bacteria | Firmicutes | Clostridia | Lachnospirales | Lachnospiraceae | Stomatobaculum | Stomatobaculum |
| sp41 | Bacteria | Firmicutes | Clostridia | Lachnospirales | Lachnospiraceae | Lachnoanaerobaculum | Lachnoanaerobaculum |
| sp44 | Bacteria | Proteobacteria | Gammaproteobacteria | Pasteurellales | Pasteurellaceae | Actinobacillus | Actinobacillus |
| sp49 | Bacteria | Bacteroidota | Bacteroidia | Bacteroidales | Prevotellaceae | Prevotellaceae YAB2003 group | Prevotellaceae YAB2003 group |
| sp50 | Bacteria | Proteobacteria | Gammaproteobacteria | Burkholderiales | Burkholderiaceae | Lautropia | Lautropia |
| sp51 | Bacteria | Firmicutes | Clostridia | Peptostreptococcales-Tissierellales | Anaerovoracaceae | NA | Anaerovoracaceae |
| sp52 | Bacteria | Firmicutes | Negativicutes | Veillonellales-Selenomonadales | Selenomonadaceae | Selenomonas | Selenomonas |
| sp55 | Bacteria | Firmicutes | Clostridia | Lachnospirales | Lachnospiraceae | Johnsonella | Johnsonella |
| sp63 | Bacteria | Proteobacteria | Gammaproteobacteria | Pasteurellales | Pasteurellaceae | Aggregatibacter | Aggregatibacter |
| sp69 | Bacteria | Firmicutes | Clostridia | Peptostreptococcales-Tissierellales | Peptostreptococcaceae | Filifactor | Filifactor |
| sp70 | Bacteria | Bacteroidota | Bacteroidia | Flavobacteriales | Weeksellaceae | Bergeyella | Bergeyella |
| sp71 | Bacteria | Patescibacteria | Saccharimonadia | Saccharimonadales | Saccharimonadaceae | NA | Saccharimonadaceae |
| sp72 | Bacteria | Actinobacteriota | Actinobacteria | Corynebacteriales | Corynebacteriaceae | Corynebacterium | Corynebacterium |
| sp77 | Bacteria | Patescibacteria | Saccharimonadia | Saccharimonadales | Saccharimonadaceae | Candidatus Saccharimonas | Candidatus Saccharimonas |
| sp82 | Bacteria | Proteobacteria | Gammaproteobacteria | Burkholderiales | Comamonadaceae | Acidovorax | Acidovorax |
| sp84 | Bacteria | Bacteroidota | Bacteroidia | Flavobacteriales | Flavobacteriaceae | Capnocytophaga | Capnocytophaga |
| sp85 | Bacteria | Actinobacteriota | Actinobacteria | Actinomycetales | Actinomycetaceae | F0332 | F0332 |
| sp87 | Bacteria | Proteobacteria | Gammaproteobacteria | Pasteurellales | Pasteurellaceae | Mannheimia | Mannheimia |
| sp90 | Bacteria | Bacteroidota | Bacteroidia | Bacteroidales | Tannerellaceae | Tannerella | Tannerella |
| sp92 | Bacteria | Firmicutes | Clostridia | Peptococcales | Peptococcaceae | Peptococcus | Peptococcus |
| sp95 | Bacteria | Patescibacteria | Saccharimonadia | Saccharimonadales | NA | NA | Saccharimonadales |
| sp98 | Bacteria | Patescibacteria | Gracilibacteria | Absconditabacteriales (SR1) | NA | NA | Absconditabacteriales (SR1) |
| sp102 | Bacteria | Firmicutes | Clostridia | Lachnospirales | Lachnospiraceae | Butyrivibrio | Butyrivibrio |
| sp104 | Bacteria | Proteobacteria | Gammaproteobacteria | Burkholderiales | Neisseriaceae | Eikenella | Eikenella |
| sp108 | Bacteria | Actinobacteriota | Actinobacteria | Bifidobacteriales | Bifidobacteriaceae | Scardovia | Scardovia |
| sp109 | Bacteria | Bacteroidota | Bacteroidia | Bacteroidales | Paludibacteraceae | F0058 | F0058 |
| sp114 | Bacteria | Fusobacteriota | Fusobacteriia | Fusobacteriales | Leptotrichiaceae | Oceanivirga | Oceanivirga |
| sp115 | Bacteria | Firmicutes | Negativicutes | Veillonellales-Selenomonadales | Selenomonadaceae | Centipeda | Centipeda |
| sp118 | Bacteria | Campilobacterota | Campylobacteria | Campylobacterales | Campylobacteraceae | Campylobacter | Campylobacter |
| sp122 | Bacteria | Firmicutes | Clostridia | Lachnospirales | Lachnospiraceae | Catonella | Catonella |
| sp125 | Bacteria | Proteobacteria | Gammaproteobacteria | Burkholderiales | Comamonadaceae | Comamonas | Comamonas |
| sp126 | Bacteria | Proteobacteria | Gammaproteobacteria | Pseudomonadales | Moraxellaceae | Enhydrobacter | Enhydrobacter |
| sp127 | Bacteria | Proteobacteria | Gammaproteobacteria | Burkholderiales | Comamonadaceae | Aquabacterium | Aquabacterium |
| sp132 | Bacteria | Firmicutes | Bacilli | RF39 | NA | NA | RF39 |
| sp136 | Bacteria | Actinobacteriota | Actinobacteria | Bifidobacteriales | Bifidobacteriaceae | Alloscardovia | Alloscardovia |
| sp141 | Bacteria | Firmicutes | Bacilli | Lactobacillales | Lactobacillaceae | Lactobacillus | Lactobacillus |
| sp147 | Bacteria | Desulfobacterota | Desulfobulbia | Desulfobulbales | Desulfobulbaceae | Desulfobulbus | Desulfobulbus |
| sp150 | Bacteria | Firmicutes | Clostridia | Peptostreptococcales-Tissierellales | Peptostreptococcaceae | Peptoanaerobacter | Peptoanaerobacter |
| sp153 | Bacteria | Firmicutes | Clostridia | Lachnospirales | Defluviitaleaceae | Defluviitaleaceae UCG-011 | Defluviitaleaceae UCG-011 |
| sp161 | Bacteria | Proteobacteria | Gammaproteobacteria | Burkholderiales | Comamonadaceae | Ottowia | Ottowia |
| sp162 | Bacteria | Firmicutes | Clostridia | Peptostreptococcales-Tissierellales | Peptostreptococcaceae | NA | Peptostreptococcaceae |
| sp163 | Bacteria | Spirochaetota | Spirochaetia | Spirochaetales | Spirochaetaceae | Treponema | Treponema |
| sp169 | Bacteria | Firmicutes | Bacilli | Mycoplasmatales | Mycoplasmataceae | Mycoplasma | Mycoplasma |
| sp170 | Bacteria | Proteobacteria | Gammaproteobacteria | Burkholderiales | Neisseriaceae | Kingella | Kingella |
| sp175 | Bacteria | Actinobacteriota | Actinobacteria | Bifidobacteriales | Bifidobacteriaceae | Bifidobacterium | Bifidobacterium |
| sp205 | Bacteria | Proteobacteria | Gammaproteobacteria | Cardiobacteriales | Cardiobacteriaceae | Cardiobacterium | Cardiobacterium |
| sp206 | Bacteria | Patescibacteria | Gracilibacteria | JGI 0000069-P22 | NA | NA | JGI 0000069-P22 |
| sp210 | Bacteria | Firmicutes | Negativicutes | Veillonellales-Selenomonadales | Veillonellaceae | Dialister | Dialister |
| sp211 | Bacteria | Actinobacteriota | Actinobacteria | Bifidobacteriales | Bifidobacteriaceae | Parascardovia | Parascardovia |
| sp215 | Bacteria | Proteobacteria | Alphaproteobacteria | Sphingomonadales | Sphingomonadaceae | Sphingobium | Sphingobium |
| sp222 | Bacteria | Firmicutes | Clostridia | Eubacteriales | Eubacteriaceae | Pseudoramibacter | Pseudoramibacter |
| sp227 | Bacteria | Bacteroidota | Bacteroidia | Bacteroidales | Prevotellaceae | Alloprevotella | Alloprevotella |
| sp238 | Bacteria | Firmicutes | Bacilli | Erysipelotrichales | Erysipelotrichaceae | Bulleidia | Bulleidia |
| sp252 | Bacteria | Proteobacteria | Gammaproteobacteria | Legionellales | Legionellaceae | Legionella | Legionella |
| sp262 | Bacteria | Firmicutes | Bacilli | Acholeplasmatales | Acholeplasmataceae | DMI | DMI |
| sp272 | Bacteria | Bacteroidota | Bacteroidia | Sphingobacteriales | Lentimicrobiaceae | Lentimicrobium | Lentimicrobium |
| sp278 | Bacteria | Actinobacteriota | Actinobacteria | Actinomycetales | Actinomycetaceae | Mobiluncus | Mobiluncus |
| sp282 | Bacteria | Firmicutes | Bacilli | Staphylococcales | Staphylococcaceae | Staphylococcus | Staphylococcus |
| sp293 | Bacteria | Proteobacteria | Gammaproteobacteria | Burkholderiales | Neisseriaceae | Conchiformibius | Conchiformibius |
| sp311 | Bacteria | Synergistota | Synergistia | Synergistales | Synergistaceae | Fretibacterium | Fretibacterium |
| sp315 | Bacteria | Bacteroidota | Bacteroidia | Bacteroidales | Prevotellaceae | NA | Prevotellaceae |
| sp318 | Bacteria | Firmicutes | Clostridia | Lachnospirales | Lachnospiraceae | Shuttleworthia | Shuttleworthia |
| sp331 | Bacteria | Firmicutes | Negativicutes | Veillonellales-Selenomonadales | Veillonellaceae | NA | Veillonellaceae |
| sp345 | Bacteria | Proteobacteria | Gammaproteobacteria | Burkholderiales | Rhodocyclaceae | Methyloversatilis | Methyloversatilis |
| sp382 | Bacteria | Patescibacteria | Saccharimonadia | Saccharimonadales | Saccharimonadaceae | GTL1 | GTL1 |
| sp411 | Bacteria | Firmicutes | Clostridia | Peptostreptococcales-Tissierellales | Peptostreptococcales-Tissierellales fa | W5053 | W5053 |
| sp416 | Bacteria | Verrucomicrobiota | Verrucomicrobiae | Verrucomicrobiales | NA | NA | Verrucomicrobiales |
| sp440 | Bacteria | Proteobacteria | Alphaproteobacteria | Caulobacterales | Caulobacteraceae | Phenylobacterium | Phenylobacterium |
| sp468 | Bacteria | Firmicutes | Clostridia | Peptostreptococcales-Tissierellales | Anaerovoracaceae | Family XIII UCG-001 | Family XIII UCG-001 |
| sp484 | Bacteria | Proteobacteria | Alphaproteobacteria | Caulobacterales | Caulobacteraceae | Brevundimonas | Brevundimonas |
| sp514 | Bacteria | Desantisbacteria | NA | NA | NA | NA | Desantisbacteria |
| sp540 | Bacteria | Bacteroidota | Bacteroidia | Flavobacteriales | Weeksellaceae | Cloacibacterium | Cloacibacterium |
| sp553 | Bacteria | Firmicutes | Bacilli | Erysipelotrichales | Erysipelotrichaceae | Erysipelotrichaceae UCG-006 | Erysipelotrichaceae UCG-006 |
| sp581 | Bacteria | Firmicutes | Bacilli | Lactobacillales | Carnobacteriaceae | Isobaculum | Isobaculum |
| sp600 | Bacteria | Actinobacteriota | Coriobacteriia | Coriobacteriales | Eggerthellaceae | Slackia | Slackia |
| sp669 | Bacteria | Bacteroidota | Bacteroidia | Bacteroidales | Porphyromonadaceae | NA | Porphyromonadaceae |
| sp703 | Bacteria | Proteobacteria | Gammaproteobacteria | Burkholderiales | Neisseriaceae | Stenoxybacter | Stenoxybacter |
| sp718 | Bacteria | Firmicutes | Negativicutes | Veillonellales-Selenomonadales | Veillonellaceae | Anaeroglobus | Anaeroglobus |
| sp742 | Bacteria | Actinobacteriota | Actinobacteria | Propionibacteriales | Propionibacteriaceae | Pseudopropionibacterium | Pseudopropionibacterium |
| sp743 | Bacteria | Proteobacteria | Gammaproteobacteria | Pasteurellales | Pasteurellaceae | Muribacter | Muribacter |
| sp754 | Bacteria | Proteobacteria | Alphaproteobacteria | Sphingomonadales | Sphingomonadaceae | Novosphingobium | Novosphingobium |
| sp822 | Bacteria | Firmicutes | Clostridia | Lachnospirales | Lachnospiraceae | NA | Lachnospiraceae |
| sp840 | Archaea | Aenigmarchaeota | Aenigmarchaeia | Aenigmarchaeales | Aenigmarchaeales fa | Candidatus Aenigmarchaeum | Candidatus Aenigmarchaeum |
| sp844 | Bacteria | Firmicutes | Clostridia | Lachnospirales | Lachnospiraceae | Howardella | Howardella |
| sp869 | Archaea | Nanoarchaeota | Nanoarchaeia | Woesearchaeales | SCGC AAA286-E23 | NA | SCGC AAA286-E23 |
| sp1024 | Bacteria | Proteobacteria | Gammaproteobacteria | Pasteurellales | Pasteurellaceae | Necropsobacter | Necropsobacter |
| sp1105 | Bacteria | Bacteroidota | Bacteroidia | Bacteroidales | Rikenellaceae | Rikenellaceae RC9 gut group | Rikenellaceae RC9 gut group |
| sp1648 | Bacteria | Proteobacteria | Gammaproteobacteria | Pseudomonadales | Pseudomonadaceae | Pseudomonas | Pseudomonas |
